# Recent Advances in Second Near-Infrared Region (NIR-II) Fluorophores and Biomedical Applications

**DOI:** 10.3389/fchem.2021.750404

**Published:** 2021-10-18

**Authors:** Yingying Chen, Liru Xue, Qingqing Zhu, Yanzhi Feng, Mingfu Wu

**Affiliations:** Department of Gynecology, Tongji Hospital, Tongji Medical College of Huazhong University of Science and Technology, Wuhan, China

**Keywords:** NIR-II, fluorophores, fluorescence imaging, biomedical applications, biological imaging

## Abstract

Fluorescence imaging technique, characterized by high sensitivity, non-invasiveness and no radiation hazard, has been widely applicated in the biomedical field. However, the depth of tissue penetration is limited in the traditional (400–700 nm) and NIR-I (the first near-infrared region, 700–900 nm) imaging, which urges researchers to explore novel bioimaging modalities with high imaging performance. Prominent progress in the second near-infrared region (NIR-II, 1000–1700 nm) has greatly promoted the development of biomedical imaging. The NIR-II fluorescence imaging significantly overcomes the strong tissue absorption, auto-fluorescence as well as photon scattering, and has deep tissue penetration, micron-level spatial resolution, and high signal-to-background ratio. NIR-II bioimaging has been regarded as the most promising *in vivo* fluorescence imaging technology. High brightness and biocompatible fluorescent probes are crucial important for NIR-II *in vivo* imaging. Herein, we focus on the recently developed NIR-II fluorescent cores and their applications in the field of biomedicine, especially in tumor delineation and image-guided surgery, vascular imaging, NIR-II-based photothermal therapy and photodynamic therapy, drug delivery. Besides, the challenges and potential future developments of NIR-II fluorescence imaging are further discussed. It is expected that our review will lay a foundation for clinical translation of NIR-II biological imaging, and inspire new ideas and more researches in this field.

## Introduction

**FIGURE 1:**
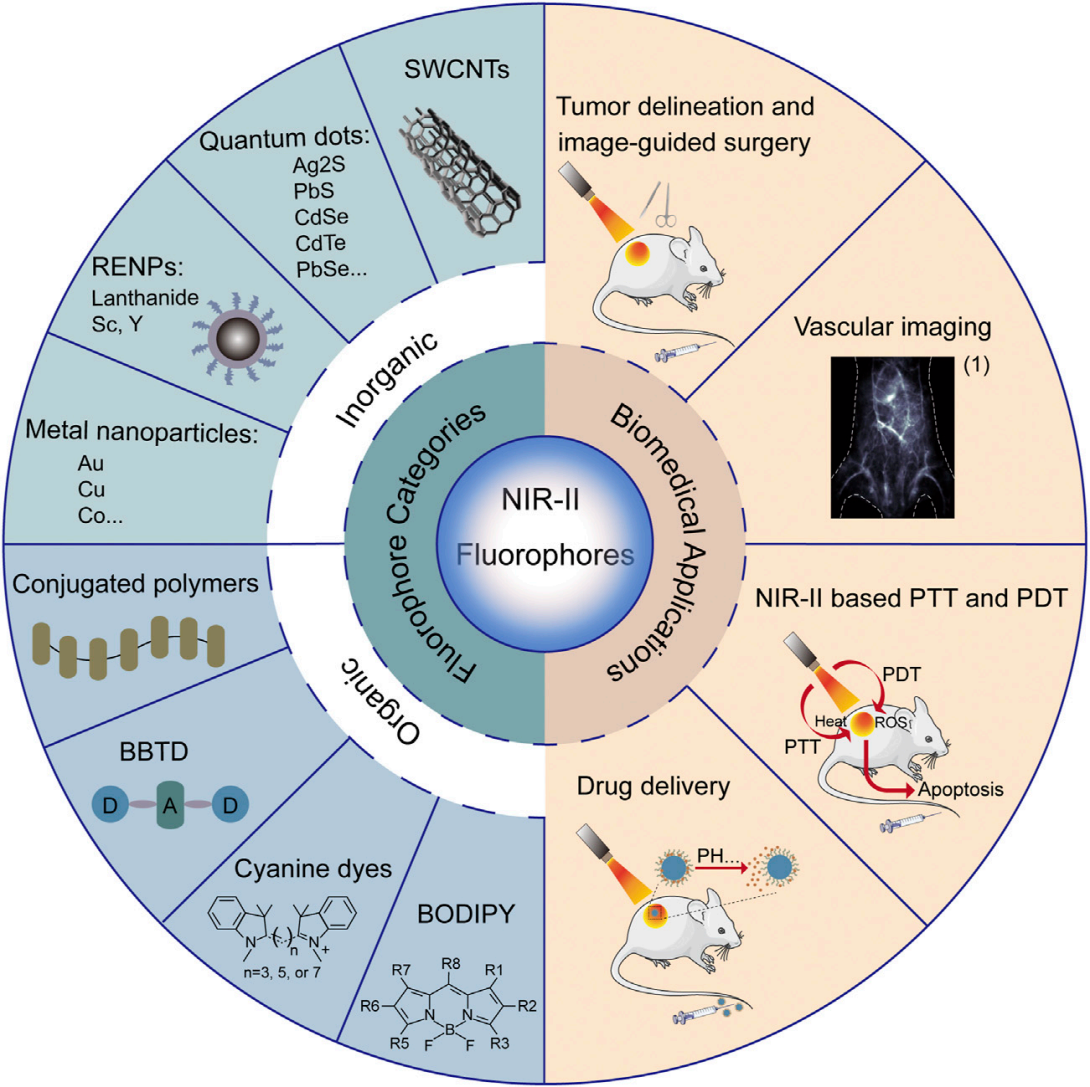
Summary of categories and biomedical applications of NIR-II fluorophores. (1) Reproduced from (Li et al., 2020) with permission from Springer Nature.

Optical imaging has the advantages of safety, no radiotoxicity, non-invasiveness, high rapid output, low detection limit and high resolution compared with other imaging modalities, such as positron emission tomography (PET), computed tomography (CT), magnetic resonance imaging (MRI) and ultrasound imaging (Hemmer et al., 2016; Zhang R. R. et al., 2017). The fluorescence imaging has promising application prospects in biomolecular detection, drug distribution and metabolism, image-guided surgery, clinical diagnosis and therapy (Hilderbrand and Weissleder, 2010). The penetration depth of photons, which is primarily determined by the absorption and scatteration of tissues, is essential to the quality of *in vivo* biological imaging (Weissleder, 2001). Since it absorbs and scatters less in biological tissues, the near-infrared light can achieve high penetration efficiency. Therefore, the fluorescence imaging technology has mainly focused on the near-infrared window (Harrison et al., 2015; Yang et al., 2016; Liu et al., 2017). However, the imaging quality of deep tissues is quite poor in the first near-infrared region (NIR-I, 700–900 nm), with the penetration depth being only 1–6 mm (Bashkatov et al., 2005). Recent studies have shown that biological imaging in the second near-infrared region (NIR-II, 1,000–1700 nm) can achieve higher penetration depth (up to 20 mm) and spatial-temporal resolution, so as to obtain a better image quality (Wang et al., 2014; Li and Pu, 2018). With the development of chemical synthesis, there have been several fluorescent probes employed for NIR-II biomedical imaging, including single-walled carbon nanotubes (SWCNTs), small organic molecules, rare-earth-doped nanoparticles (RENPs), quantum dots (QDs), conjugated polymers and other inorganic nanoparticles (Antaris et al., 2016; Cai et al., 2019; Chen et al., 2020; Du et al., 2020; Mandal et al., 2020; Yu et al., 2020; Chen et al., 2021). In this paper, the recent developed fluorescent probes for NIR-II imaging and their biomedical applications are reviewed (shown in Figure 1) and prospected, in order to promote clinical translation and inspire new ideas for the development of NIR-II fluorescent imaging technology.

## Categories of NIR-II Fluorescent Probes

### Inorganic NIR-II Fluorophores

Compared to organic NIR-II fluorophores, inorganic NIR-II nanoprobes have relatively high quantum yields and low photobleaching sensitivity, offering unique advantages in areas such as liver, kidney, brain and lung imaging.

#### Single-Walled Carbon Nanotubes

SWCNTs are about 1 nm in diameter and can be hundreds to thousands of nanometers in length, and can be dissolved in aqueous solution with the help of dispersants. SWCNTs have a large Stokes shift, good photostability and fluorescence emission in the near-infrared band, and have long been used as optical biosensors. Due to their inherent broad NIR-II fluorescence emission, SWCNTs are increasingly used for *in vivo* imaging (Ding et al., 2019).

Welsher et al. applied SWCNTs as the first-generation NIR-II fluorescent probe for biomedical fluorescent imaging and obtained deeper tissue penetration and higher temporal-spatial resolution (Welsher et al., 2009). To overcome the potential toxicity of SWCNTs, which is a major obstacle to clinical translation, Tsukasa et al. used phospholipid polyethylene glycol to encapsulate oxygen-doped SWCNTs to obtain o-SWCNT-PEG. o-SWCNT-PEG is a biocompatible probe that effectively overcomes the toxicity of SWCNTs and can be used for vascular imaging (Takeuchi et al., 2019).

#### Quantum Dots

QDs have drawn much attention due to their broad excitation spectrum, narrow emission spectrum, high quantum yield, resistance to photobleaching, and high temporal-spatial resolution. Moreover, the pharmacokinetics and tissue distribution of quantum dots such as PbS, CdSe and Ag_2_S can be adjusted by fine-tuning the size and shape of the dots (Matea et al., 2017).

QDs for NIR-II imaging are mainly group Ⅱ-Ⅳ and Ⅳ-Ⅵ semiconductor materials, such as CdSe, CdTe and PbSe, but the heavy metal elements contained in them greatly limit their subsequent biomedical applications (Matea et al., 2017). Therefore, the development of new NIR-II fluorescent QDs with good biocompatibility and efficient luminescence is a hot and difficult research area. Ag_2_S QDs have been widely used in near infrared imaging because of their low toxicity, which is lower than other quantum dots containing Pb, Cd, Te, Se. Besides, the fluorescence can be tuned from 687 to 1,294 nm. It also has high fluorescence stability and small particle size (Zhao and Song, 2014). Hong et al. first used Ag_2_S in NIR-II imaging and non-specific tumor detection (Hong et al., 2012). Protein nanocage (PNC) encaged Ag_2_S QDs could track the immigration behavior of PNC *in vivo* Li et al. (2015). successfully synthesized graphene quantum dots with strong absorbance at NIR-II and discussed their potential biomedical applications in tumor photothermal therapy (Liu et al., 2020).

#### Rare-earth-doped Nanoparticles

RENPs have received increasing attention due to their large Stokes shifts, minimal photobleaching, narrow and multi-peak emission properties, and negligible excitation-emission band overlap. Moreover, due to the tunable emission wavelength and extended luminescence lifetime by doping with different rare-earth metal ions, RENPs have become a research hotspot for NIR-II imaging and have a wide range of application prospects (Yu et al., 2020).

Due to the long fluorescence lifetimes and large Stokes shifts, lanthanide RENPs are widely used as fluorescent probes. Recently, Li et al. used liposome-encapsulated NIR-II lanthanide fluorophore RENPs to obtain RENPs@Lips with double emission wavelength at 1,064 nm and 1,345 nm and large Stokes shifts. RENPs@Lips have favorable biocompatibility, good intravenous excretion and excellent photochemical properties, which are suitable for preclinical evaluation and monitoring of physiological and pathological processes and facilitate their future clinical translation (Li D. et al., 2019). Rare Earth element Yb/Er co-doped nanoparticles (ErRENPs) have been reported to have luminescence properties at 1,530 nm and exhibit large Stokes shifts, long lifetime, and good photostability, which are considered as favorable candidates for a new generation of near-infrared probes. However, Er^3+^ ions are prone to energy transfer to the nanocrystal surface, leading to severe luminescence bursts. Cao et al. used a Nd^3+^-sensitized Yb^3+^ system to transfer energy to the luminescence center Er^3+^ with the assistance of Ce^3+^ internal ions. By introducing polyethylene glycol ligands, the water solubility of the nanoparticles was increased, and a large blood circulation time was achieved. Finally, the acquisition of NIR-II fluorescence signals from water-soluble nanoprobes can be applied for high-resolution tracking and imaging of tumors (Cao et al., 2020).

#### Metal Nanoparticles

The inert metal-based emitters are well suited for NIR-II imaging because they are less likely to cause fluorescence bursts. Gold nanoclusters (Au NCs) are a typical example. Au NCs have many special advantages such as good biocompatibility, smaller size than the renal excretion threshold, good photostability, easy modification, excellent photothermal activity, making them promising new NIR-II probes (Wu et al., 2020). In addition to their NIR-II imaging capabilities, Au NCs with bare gold atoms can react with certain sulfhydryl-containing species such as glutathione (GSH) by forming gold-sulfur covalent bonds. Yang et al. developed a bifunctional pyrophotometric nanodrug (Au NCs-Pt) using Au NCs to deliver Pt. On the one hand, the NIR-II imaging capability of Au NCs-Pt ensures effective visualization of Pt transport in a high-resolution deep tumor model; on the other hand, Au NCs-Pt binds GSH via Au-S bond to scavenge intracellular GSH and thus effectively sensitize tumor cells to platinum drugs. Therefore, Au NCs-Pt can be used as a therapeutically integrated nanomedicine to maximize the use of platinum-dependent chemotherapy while monitoring platinum transport in deep tissues through high-resolution NIR-II imaging (Yang Y. et al., 2020).

Intermetallic compounds or alloys formed by mixing various metallic elements can greatly expand the properties of metals. Alloy nanoparticles have attracted much attention because its chemical and physical properties vary with composition, atom distribution and particle size. The new functions of alloy particles can be developed through the introduction of another metal. For example, Andolina et al. introduced copper into Au nanodots to form Au/Cu alloy particles. The fluorescence of Au/Cu nanoparticles gradually shifted from NIR-I to NIR-II by adjusting the content of copper in the alloy particles (Andolina et al., 2013). Marbella et al. further introduced Co. into Au nanodots to form Au/Co. alloy particles, which had both magnetic and near infrared fluorescence tunable functions (Marbella et al., 2014). These multi-functional alloy nanoparticles have wide application in the field of biomedical imaging.

### Organic NIR-II Fluorophores

Most fluorescent probes are excreted slowly and are mainly retained in the spleen and liver (Zhang et al., 2013). Organic NIR-II fluorophores have a well-defined chemical structure and are easily metabolizable, low-toxic and biocompatible, making them attractive and promising for future clinical applications.

#### Conjugated Polymers

The electron-rich donor and electron-absorbing acceptor can make the band gap of the copolymer small, so the conjugated polymer generated by alternating D-A copolymerization has the advantages of small band gap and easy adjustment, which is an effective way to design NIR-II probes. Semiconductor polymer dots (Pdots) are a new type of organic fluorescent material that has emerged in recent years. Compared with conventional fluorescent dyes, Pdots have broad absorption, symmetric narrow emission, high luminance, high photostability and large Stokes shift (Chen et al., 2021). Therefore, nanoparticles composed of highly fluorescent semiconductor polymers are considered as an effective fluorescent probe and show promising applications in bioimaging, molecular detection, and guiding drug therapy.

Semiconductor polymers in the form of nanoparticles usually exhibit fluorescence burst. The bursts can be attributed to strong inter-chain π-π stacking interactions, leading to the formation of non-emitting exciton and radical complexes. Recently, Zhang et al. proposed a dual fluorescence enhancement mechanism to enhance the NIR-II fluorescence of Pdots. In this study, the aggregation-induced emission properties of phenothiazine units were exploited to reduce the nonradiative decay paths of aggregated polymers on the one hand, and a large number of side chain groups were introduced to further enhance the fluorescence quantum yield by reducing the strong inter-chain π-π stacking interactions through spatial site resistance on the other hand (Zhang Z. et al., 2020). This dual enhancement strategy has potential applications in the design of NIR-II fluorophores for *in vivo* fluorescence imaging.

#### Benzobisthiadiazole

Fluorophores with donor-acceptor-donor (D-A-D) characteristics, such as BBTD derivatives, have large Stokes shifts and high imaging quality. The spatial structure of the strong electron donor and the central electron acceptor in the D-A-D scaffold can narrow the energy gap between the highest occupied molecular orbital (HOMO)/the lowest unoccupied molecular orbital (LUMO) energy levels of the hybridization and red-shift the fluorescence emission to the NIR-II region (Zhou et al., 2018). The absorption and emission spectra can be effectively modified by adjusting the acceptor and donor structures of the D-A-D fluorophore.

CH1055 is the first reported D-A-D type organic NIR-II fluorophore for *in vivo* biomedical imaging. CH1055 performed excellently in pharmacokinetics and approximately 90% of them was excreted quickly through the renal system within 24 h (Antaris et al., 2016). Generally, the maximum excitation and emission wavelengths of BBTD-based fluorophores are approximately 800 and 1,000 nm, respectively. The design of new fluorophores with longer wavelengths will facilitate the imaging of deep tissues in NIR-II. It’s reported that replacing the S atom in the BBTD backbone with Se can red-shift the emission wavelength. In addition, the introduction of electron-giving amino groups can also extend the emission wavelength to NIR-II. Recently, Fang et al. developed a new organic small molecule fluorophore FM1210 with a maximum emission wavelength of 1,210 nm by introducing both a Se atom and an amino group into the BBTD backbone. The quantum yield and brightness of FM1210 is greatly enhanced and the imaging quality is significantly improved (Fang et al., 2020).

#### Cyanine Dyes

Polymethylene skeleton-based anthocyanine dyes contain an extended π-cinjugation system and have a unique conjugated skeleton structure. By lengthening the polymethylene chain, increasing the donor strength of the heterocyclic ring, or changing the heteroatom from oxygen to other sulfur elements, the dyes can be red-shifted in absorption wavelength. Compared to D-A-D dyes, cyanine dyes are relatively simple to synthesize and have high absorption intensity, especially for near-infrared light, making them suitable for near-infrared imaging (Schnermann, 2017; Sun et al., 2019).

Currently, NIR-II cyanine dyes have disadvantages in bioimaging, such as poor stability, small Stokes shift or solvent burst. To address these problems, Ren et al. proposed a new idea of constructing NIR-II fluorescent probes by increasing the spatial resistance and electronic asymmetry, and developed a series of stable, high quantum yield, and solvent burst resistant elite fluorophores (NIR II-RTs). In addition, due to the introduction of the carboxylic acid functional group, the new dye NIR II-RT3/4 can generate a powerful fluorescence switching mechanism through helical cyclization, and thus the NIR II-RT dye can be designed as an activatable NIR-II fluorescent probe (Ren et al., 2020).

#### Boron Dipyrromethene

BODIPY dyes have high quantum yields, excellent chemical and photophysical stability, and play a vital role in molecular imaging and drug delivery. The classical BODIPY absorbs in the range of 500–600 nm and has a rather small Stokes shift. Based on the strong electron-absorbing nature of BODIPY, the introduction of electron-giving groups can induce absorption and emission wavelength redshifts (Bai et al., 2019; Zhang J. et al., 2020). In recent years, NIR-II organic fluorescent materials based on BODIPY have also been developed rapidly.

The poor water solubility of aza-BODIPYs has limited their application in *vivo* studies. Sancey et al. reported a new strategy to prepare water-soluble aza-BODIPYs, named SWIR-WAZABY-01, by introducing ammonium groups on boron atoms. So SWIR-WAZABY-01 can be used for NIR-II imaging of tumors without hydrophilic encapsulation or PEG assistance. The aza-BODIPY-based dyes can rapidly reach and accumulate in tumors and remain *in vivo* for up to 1 week (Godard et al., 2020). Bai et al. developed a new class of aza-BODIPY dyes: NJ960, NJ1030 and NJ1060, which can redshift the NIR emission to NIR-II. In addition, these dyes have good photophysical properties, such as large Stokes shift, good photostability and high fluorescence brightness in aqueous solution. The *in vivo* NIR-II fluorescence imaging results demonstrate the high resolution and deep penetration imaging capability of NJ1060 (Bai et al., 2019).

## Applications of NIR-II Biomedical Imaging

### Tumor Delineation and Image-Guided Surgery

Surgical resection of solid tumors is currently the most effective treatment strategy for cancer, and the removal of microscopic tumors and metastatic lesions during surgery is an effective way to reduce tumor recurrence. Currently, the common methods used by oncologic surgeons are visual observation, empirical judgment by hand touch, rapid pathological sectioning, intraoperative ultrasound, optical imaging and other auxiliary means, but all of them can hardly meet the demand for comprehensive detection of intraoperative microscopic tumor lesions. It is particularly important to develop new multimodal and multiscale imaging techniques and multifunctional contrast agents for precise tumor identification and surgical navigation.

**FIGURE 2:**
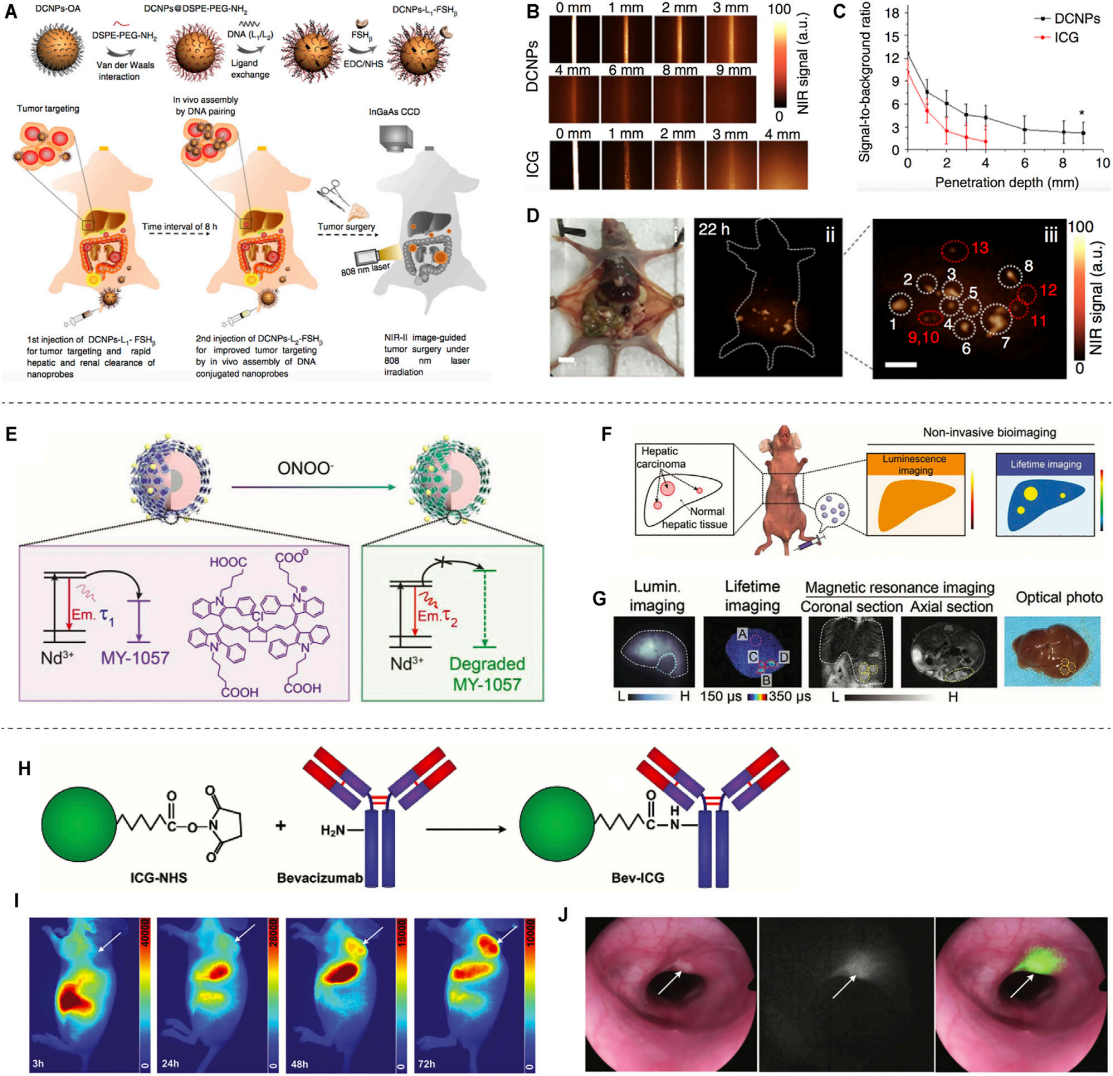
NIR-II based Tumor delineation and image-guided surgery. **(A)** Schematic illustration of DNA and FSH_β_ modified DCNPs and *in vivo* assembly of DCNPs with improved tumor navigation and excision. **(B)** The penetration depth of DCNPs and ICG of phantom tissues. **(C)** Signal to background ratio of DCNPs and ICG. **(D)** The NIR-II nanoparticles labeled peritoneal metastatic tumors (reproduced from (Wang et al., 2018) with permission from Springer Nature). **(E)** In the presence of ONOO^−^, MY-1057 degrades sensitively, leading to the lifetime recovery of NIR-II region. **(F)** Illustration of NIR-II luminescence and lifetime imaging for hepatocellular carcinomas. **(G)** Noninvasive luminescence imaging, lifetime imaging and MRI of hepatocellular carcinomas, and optical photo of the dissected liver (reproduced from (Zhao et al., 2020) with permission from John Wiley and Sons). **(H)** Synthesis process of Bev-ICG. **(I)** Whole body NIR-II fluorescence imaging of mice after injection of Bev-ICG. **(J)** Simultaneous white-light and fluorescence images of tumors (reproduced from (Suo et al., 2019) with permission from John Wiley and Sons).

Based on the distinctive technical features of NIR-II imaging, it has a promising application in surgical tumor navigation. For example, previous studies reported on fluorescent molecular dyes-mediated tumor and lymphadenectomy (Antaris et al., 2016; Sun et al., 2017; Sun et al., 2018). Professor Zhang synthesized the NIR-II emitting downconversion nanoparticles (DCNPs) modified with DNA and targeting peptides-FSH_β_ for enhanced surgical navigation of metastatic ovarian cancer (Figure 2A). It is proved that the DCNPs based imaging has good photostability and deep tissue penetration (8 mm), which is superior to that of clinical approved ICG (Figures 2B,C). Furthermore, the metastases with <1 mm can be completely excised under NIR-II bioimaging guidance (Figure 2D) (Wang et al., 2018). Recently, Zhang’s group synthesized a tumor-microenvironment (ONOO^−^) responsive NIR-II dye, MY-1057-the acceptor of FRET, to detect hepatocellular carcinomas (Figures 2E,F). It can accurately detect single and multiple hepatocellular carcinomas through lifetime-based imaging (Figure 2G) (Zhao et al., 2020). However, since no clinically approved NIR-II fluorescent dyes have been reported, no new probes have been used for NIR-II clinical surgical navigation. The NIR-I fluorescent dye indocyanine green (ICG) is an FDA-approved probe molecule for clinical surgical navigation, and ICG-mediated NIR fluorescence imaging can assist doctors in detecting microscopic tumor lesions during tumor resection (Boni et al., 2015; Zhang Y.-M. et al., 2017). In recent years, it has been found that the terminal tails of the NIR-I dyes indocyanine green (ICG) and methylene blue (MB) fluorescence bands can be extended to the NIR-II band, which has facilitated the clinical translation of NIR-II imaging technology for clinical surgical navigation (Yu et al., 2019). ICG conjugated bevacizumab (Bev-ICG) is successfully constructed and evaluated along with the NIR-II endoscopy imaging system for rat colorectal cancer detection (Figures 2H–J). The tumors can be shown obviously in fluorescence imaging, while easy to miss in white-light images (Figure 2J) (Suo et al., 2019). Based on the aforementioned FDA-approved indocyanine green with NIR-II tail fluorescence and its favorable results in NIR-II imaging in animal models, these findings pave the way for clinical translation of NIR-II surgical navigation.

### Vascular Imaging

The number of people with cardiovascular and cerebrovascular diseases is increasing. Techniques commonly used in clinical angiography include nuclear magnetic resonance (NMR), CT and B-mode ultrasonography. However, these techniques are costly, have low sensitivity, and cannot be used for real-time detection. Recently, several scientists have designed NIR-II fluorophores for *in vivo* vascular imaging. These NIR-II fluorophores perform excellently and can clearly image the fine structure of capillaries.

**FIGURE 3:**
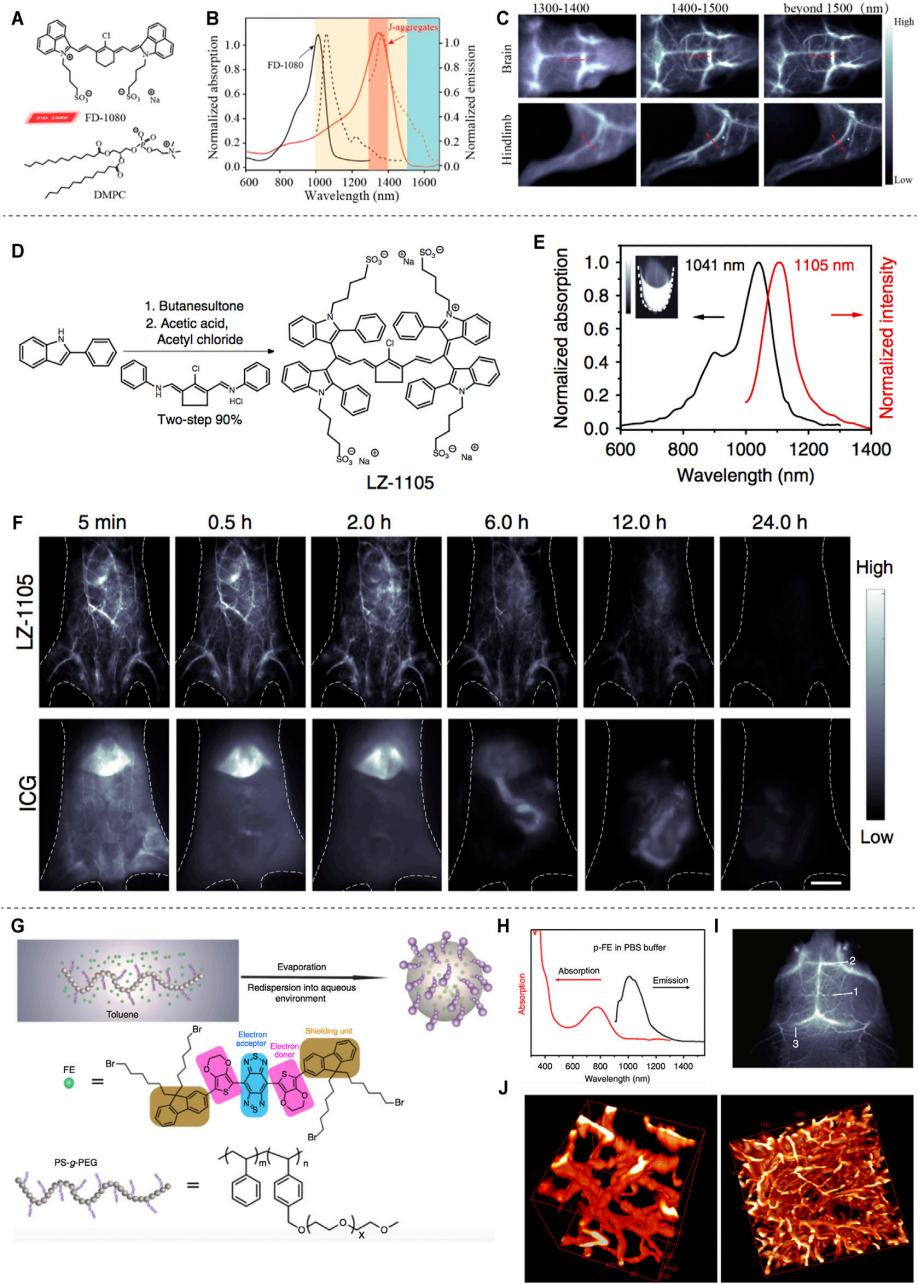
NIR-II based vascular imaging. **(A)** Structure of FD-1080 and DMPC. **(B)** Normalized absorption and emission of FD-1080 monomer and J-aggregates. **(C)** Fluorescence imaging of brain and hindlimb vessels achieved by J-aggregates (reproduced from (Sun et al., 2019) with permission from American Chemical Society). **(D)** Synthetic route of LZ-1105. **(E)** Normalized absorption and intensity of LZ-1105. **(F)** Fluorescence imaging of mice body injected with LZ-1105 (reproduced from (Li et al., 2020) with permission from Springer Nature). **(G)** Synthesis of p-FE. **(H)** Absorption and emission spectra of p-FE in PBS. **(I)** Imaging of mouse brain vessels injected with p-FE. **(J)** 3D reconstruction of vasculatures in brain (reproduced from (Wan et al., 2018) with permission from Springer Nature).

In 2019, professor Zhang reported the J-aggregates for NIR-II noninvasive dynamic vascular imaging (Figures 3A–C). The J-aggregates are formed by self-assembly of cyanine dye FD-1080 and 1,2-dimyristoyl-*sn*-glycerol-3-phosphocholine (DMPC) (Figure 3A). It showed high signal-to-background ratio and superior spatial resolution in brain and hindlimb vasculature imaging beyond 1,500 nm (Figure 3C) (Sun et al., 2019). Considering the limited blood circulation time (5–60 min) of reported fluorescent dyes, professor Zhang synthesized an organic NIR-II fluorophore (LZ-1105) with long blood half-time (3.2 h) for *in vivo* real-time dynamic vascular imaging (Figures 2D–F) (Li et al., 2020). Wan et al. developed an ultrabright NIR-II fluorophore p-FE for three-dimensional vessel imaging in the brain of mice. Benefited from both high brightness and deep penetration of the developed NIR-II fluorophore, tiny vesicles with 5–7 µm width could be visualized clearly (Figures 3G–J) (Wan et al., 2018).

### NIR-II Based Photothermal Therapy and Photodynamic Therapy

Although chemotherapy is one of the main options for clinical tumor treatment, it has the disadvantages of non-specificity and side effects. In recent years, optical therapy, mainly including NIR-II fluorophores-based photothermal therapy (PTT) and photodynamic therapy (PDT), has received much attention as a new non-toxic treatment. Under NIR-II laser irradiation, NIR-II fluorophores can not only emit fluorescence signal, but also generate heat energy or reactive oxygen species (ROS) to kill the lesion, which further demonstrates the application prospect of NIR-II fluorescent probes.

**FIGURE 4:**
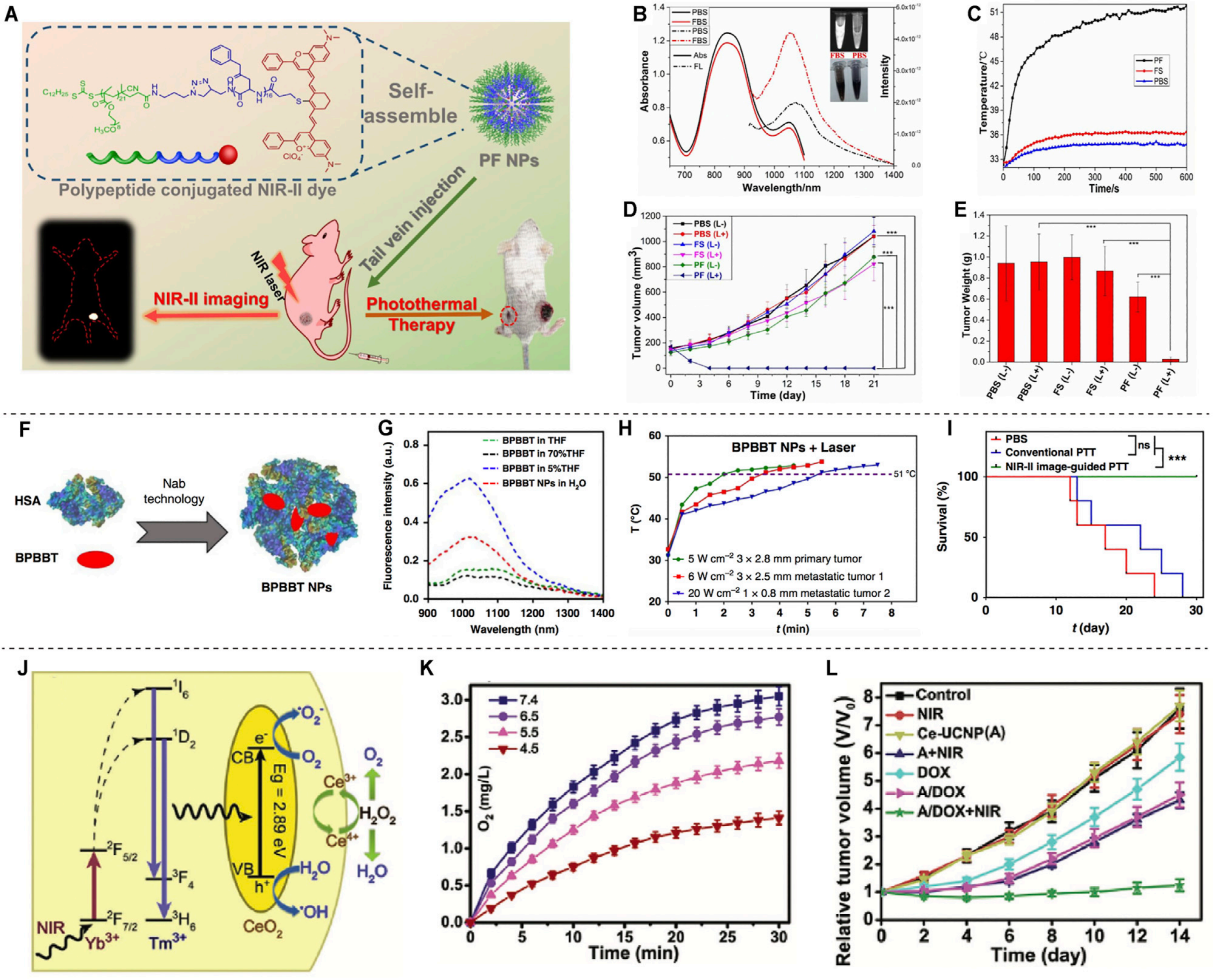
NIR-II based PTT and PDT. **(A)** Schematic illustration of the NIR image-guided PTT by PF. **(B)** Absorption and fluorescence emission spectra of PF in PBS and FBS. **(C)** The temperature profile of tumors after injection with PF under laser irradiation. **(D)** The tumor volume of the mice after PTT. **(E)** The tumor weight after PTT (reproduce from (Li et al., 2019) with permission from American Chemical Society). **(F)** Schematic illustration of BPBBT NPs. **(G)** Fluorescence intensity of BPBBT NPs in water. **(H)** Temperature change curve of tumors after treatment with BPBBT NPs. **(I)** Survival curves of mice bearing colon cancer following different treatments (reproduced from (Gao et al., 2019) with permission from Springer Nature). **(J)** Schematic illustration of the mechanism of O_2_ generation. **(K)** Producing of O_2_ by Ce-UCNPs catalysis in aqueous solution. **(L)** Tumor growth curves after various treatments (reproduced from (Yao et al., 2018) with permission from John Wiley and Sons).

PTT mainly uses photothermal reagents to convert light energy into heat energy to kill tumor cells (Zhi et al., 2020). Li et al. constructed a tumor-targeting probe PF NPs by combining the NIR-II fluorescent material Flav7 with an amphiphilic peptide (Figure 4A). It was shown that PF NPs have significant photothermal conversion efficiency, favorable photothermal stability, negligible cytotoxicity, excellent blood circulation time and tumor site enrichment, and were successfully used for NIR-II fluorescence imaging-guided PTT for tumors (Figures 4B–E) (Li T. et al., 2019). Gao et al. (2019) used human serum albumin (HSA) combined with BPBBT, a lipophilic D-A-D NIR-II fluorophores, to construct BPBBT nanoparticles (BPBBT NPs) (Figure 4F). The specific binding changes the planarity and limits the intramolecular rotation of the fluorophores, and modulates its fluorescence and photothermal conversion properties (Figure 4G). The tiny colon tumors (0.5 × 0.3 mm) can be clearly visualized in mice under the guidance of BPBBT NPs fluorescence imaging. Furthermore, BPBBT NPs allow effective photothermal ablation therapy of tumors (Figure 4H,I) (Gao et al., 2019).

For PDT, tumor cells are mainly killed by singlet oxygen (^1^O_2_) or other reactive oxygen species (ROS) produced by photosensitizers (Donohoe et al., 2019). The ultra-small Cu_2-x_Se nanoparticles (CS NPs) could generate vast amount of ROS through electron transfer and energy transfer mechanisms and show excellent PDT performance under NIR-II laser (1,064 nm) irradiation (Zhang et al., 2019). A necessary condition for effective PDT treatment is to have sufficient oxygen in tissues. While the tumor microenvironment is generally hypoxic, which is not conductive to PDT. Meanwhile, PDT will further consume oxygen, which aggravates the lack of oxygen in tumors. Therefore, PDT efficiency can be increased by increasing oxygen supply to tumor tissues, such as using perfluorocarbon and hemoglobin to deliver oxygen to tumor sites, or using catalase to decompose H_2_O_2_ produced by tumor cells to obtain O_2_ (Yang N. et al., 2020). A synergetic one-for-all mesoporous cerium oxide upconversion biophotocatalyst nanoparticles (Ce-UCNPs) were developed for hypoxia cancer PDT, which could achieve intratumorally endogenous self-sufficiency of O_2_ by decomposing H_2_O_2_ through enzymelike catalysis (Figure 4J–L) (Yao et al., 2018).

### Drug Delivery

Compared with traditional therapeutic agents, controllable drug delivery systems have many advantages, such as effective protection of bioactive drugs, high loading capacities and high therapeutic index.

**FIGURE 5:**
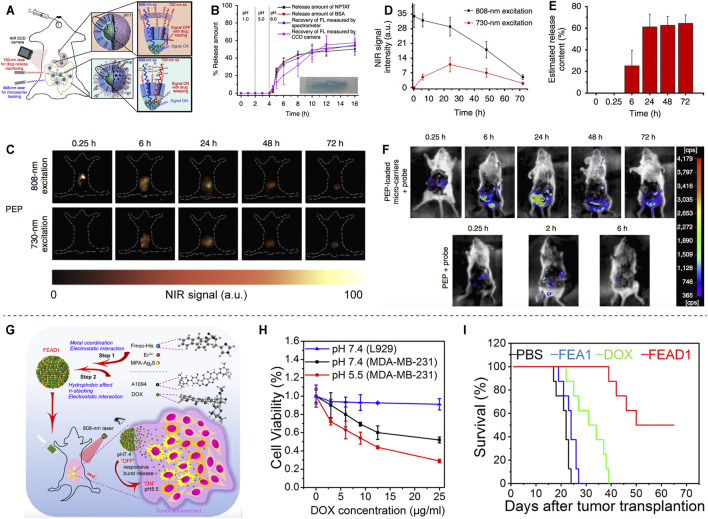
NIR-II based drug delivery. **(A)** Schematic illustration of the absorption competition-induced emission bioimaging system. **(B)** The release profiles of microcarriers in simulated gastrointestinal tract fluids. **(C)** Fluorescence imaging of mice after gavage with PEP-NPTAT-loaded microcarriers under excitation. **(D)** Signal intensity of **(C)**. **(E)** *In vivo* release percentage of PEP-NPTAT from microcarriers. **(F)** Fluorescence imaging of mice after gavage with PEP-NPTAT-loaded microcarriers and a peptide probe, and PEP molecules and a peptide probe (reproduced from (Wang et al., 2017) with permission from Springer Nature). **(G)** Schematic illustration of the synthesis of FEAD1 and capability for tumor theranostics. **(H)** Cell viability of L929 and MDA-MB-231 cells under different treatments. **(I)** Survival curves of mice following different treatments (reproduced from (Ling et al., 2020) with permission from John Wiley and Sons).

A novel lanthanide-based NIR-II fluorescent mesoporous microcarrier for protein drug delivery has been reported. The microcarrier can be tracked and the amount of drug released can be quantified by measuring the NIR-II fluorescence signals (Figure 5A). The microcarriers remain in the gastrointestinal tract for up to 72 h, with minimal deposition (<0.1%) in other non-target organs. Protein drugs show little release in neutral and acidic organs, such as stomach (PH = 1) and duodenum (PH = 5), but sustained release in alkaline intestine (PH = 8) (Figure 5B). The protein release efficiency of the oral microcarriers is up to 62% after 72 h (Figures 5C–E), and the activity of protein drugs is greatly preserved (Figure 5F) (Wang et al., 2017). NIR-II-based oral drug delivery systems offer novel design strategies for therapeutic agents. Recently, a tumor environment-activated NIR-II particle, FEAD1, is developed for precise diagnosis and therapy of peritoneal metastases (Figure 5G). FEAD1 carries the chemotherapeutic drug doxorubicin, which specifically releases at the tumor site due to the acidic tumor environment (Figure 5H). The FEAD1 shows desirable therapeutic effect on peritoneal metastases (Figure 5I) (Ling et al., 2020). This tumor environment-activated therapeutic strategy has great prospects for clinical applications in the future.

## Discussions and Perspectives

In summary, we have outlined multiple advantages of NIR-II fluorescent probes, such as high spatial-temporal resolution, deep tissue penetration, low autofluorescence and reduced photon scattering. Due to its superiorities, NIR-II fluorescence imaging is able to observe deeper anatomical structures with higher resolution, and can be used in wide-ranging biomedical practices, such as tumor delineation and image-guided surgery, vascular imaging, NIR-II-based photothermal therapy and photodynamic therapy, and drug delivery (Figure 1).

Despite the success of NIR-II fluorophores has enriched our knowledge and applications in the field of NIR-II bioimaging, current researches are mainly focused on basic study and there is still a long way to go in terms of clinical applications. Therefore, more efforts are needed to address the following issues. 1) Surgical navigation is one of the shortcuts for clinical transformation of NIR-II fluorescence imaging. As an important technical support for surgical navigation, fluorescent probes are used to determine the location of the lesion by real-time probe signals with the most intuitive image information, which can improve the surgical resection rate while minimizing the damage to normal tissue. However, current clinical approval of NIR-II fluorescent probes is limited to ICG trailing NIR-II fluorescence-mediated surgical navigation. Clinical application-oriented development of high-performance NIR-II fluorescent probes is an important driving force to break through the bottleneck of fluorophore technology and promote the clinical translation of NIR-II imaging. 2) The lymphatic system is closely related to the occurrence, development, metastasis and prognosis of tumors. ICG is used to monitor lymphatic vessels and sentinel lymph nodes in clinic. Development of probes for lymphatic system imaging has essential diagnostic, therapeutic and prognostic significance for tumors. 3) Most of the currently constructed NIR-II organic small molecule fluorescent probes are excited in the NIR-I region, but the NIR-II laser with higher safety and deeper penetration is less utilized, so the NIR-II lase-excited probes need to be further developed. Moreover, most of the developed probes have fluorescence emission tails extending to the NIR-IIb region, and the development of fluorescent probes with the main emission peak in the NIR-IIb region is a further expansion of NIR-II biomedical imaging. 4) At present, most of NIR-II fluorophores have poor biocompatibility, low water solubility and stability, slow metabolism, high toxicology, and lack of specific tissue targeting. The design and synthesis of more excellent fluorescent probes will help facilitate the application of NIR-II imaging technique in the field of biomedicine. 5) The development of multimodal imaging techniques in combination with other imaging modalities in the NIR-II biomedical imaging can achieve complementary advantages and superior outcomes, which could be more suitable for investigating biological changes and clinical applications. 6) The interaction of NIR-II fluorescent probes with biological systems of other animals (e.g. rabbits and monkeys) still needs to be further explored in order to obtain more comprehensive results on long-term biotoxicity, immune response and pharmacokinetics in animals, which will provide important support for practical clinical applications.

NIR-II fluorescence imaging is still in its infant stage. It is necessary to further develop new NIR-II fluorescent probes with tunable emission wavelengths, high quantum yields and low biological toxicity. It is expected that promising multi-functional NIR-II fluorescent probes will enable multimodal imaging and theranostics in clinical practice.
